# National Belgian Study on Terbinafine Resistance in *Trichophyton interdigitale/mentagrophytes/indotineae* (2022–2023): Epidemiology and Molecular Features

**DOI:** 10.3390/jof11090676

**Published:** 2025-09-13

**Authors:** Rosalie Sacheli, Sabrina Egrek, Khalid El Moussaoui, Bahoz Kurt, Emilie Machowski, Saadia Harag, Marie-Pierre Hayette

**Affiliations:** 1 Clinical Microbiology Department, National Reference Center for Mycoses, Center for Interdisciplinary Research on Medicines, University Hospital of Liège, 4000 Liege, Belgium; 2 Dermatopathology Department, University Hospital of St Pierre, 1000 Brussels, Belgium

**Keywords:** dermatophytes, terbinafine resistance, squalene epoxidase, epidemiology, *Trichophyton indotineae*, *Trichophyton mentagrophytes* type VII

## Abstract

**Objectives**: *Trichophyton indotineae*, a dermatophyte closely related to *T. interdigitale* and *T. mentagrophytes*, is of growing concern due to its high terbinafine resistance and widespread presence in India. Its emergence in Europe calls for enhanced surveillance. Resistance is linked to mutations in the squalene epoxidase (*SQLE*) gene. This multicentric national study aimed to evaluate the prevalence, terbinafine susceptibility, and phylogenomics of *T. interdigitale/mentagrophytes/indotineae* strains in Belgium, with a focus on SQLE substitutions. **Methods**: Between February 2022 and April 2023, 137 isolates from 16 Belgian labs were analyzed for antifungal susceptibility using the EUCAST E.Def.11.0 method. Whole-genome sequencing (WGS) was performed via Illumina sequencing method. **Results:** Phylogenomic analysis identified 8 *T. indotineae*, 91 *T. interdigitale*, and 38 *T. mentagrophytes* (including 7 genotype VII strains). Terbinafine resistance (5.1%) was mainly found in *T. indotineae* (87.5%), always linked to SQLE substitutions. *T. interdigitale* was fully susceptible. *T. mentagrophytes* showed mildly elevated MICs, often associated with K276N substitution. **Conclusions:** Terbinafine-resistant *T. indotineae* is emerging in Belgium, mostly via imported cases. Continued molecular surveillance and species-specific treatment strategies are essential.

## 1. Introduction

Dermatophytosis is a highly prevalent superficial fungal infection affecting the skin, hair, and nails, with an estimated global prevalence of 20–25% [1,2]. In recent years, concerns have been raised about the emergence of terbinafine resistance worldwide. Treating dermatophytosis with the recommended dosing and duration of conventional antifungal therapies has become increasingly challenging due to chronic, persistent infections and novel clinical presentations, such as inflammatory and pruritic forms of recalcitrant tinea cruris, extensive tinea corporis, and tinea faciei. Severe cases can progress to tinea universalis, involving the entire body [3,4]. This phenomenon was first reported in India and neighboring regions, where outbreaks have been documented since 2018 [5,6]. Terbinafine, the first-line treatment for dermatophytosis, has limited alternatives, making resistance particularly concerning. The primary resistant species is *Trichophyton indotineae*, a recently characterized species closely related to *T. interdigitale/mentagrophytes* formerly named *Trichophyton mentagrophytes* ITS genotype VIII [7]. This species shows high terbinafine resistance rates compared to *T. interdigitale/mentagrophytes* and is primarily transmitted via person-to-person contact [8]. In India, *T. indotineae* has become the predominant causative agent of tinea corporis, surpassing *T. rubrum* as the leading dermatophyte species [9,10,11]. Terbinafine resistance rates among this species depend on the geographical region but can reach 75% in some Indian parts [9]. Terbinafine resistance in this species is primarily associated with mutations in the squalene epoxidase (*SQLE*) gene, its molecular target. Various substitutions linked to resistance have already been described. Specifically, alterations in key amino acid hotspots of SQLE, including Leu393, Phe397, Phe415, and His440, have been identified, with the most common substitutions being Phe397Leu and Leu393Phe [5,12,13,14,15,16,17,18,19,20,21,22]. Due to globalization, *T. indotineae* has now been identified in numerous countries beyond India. In Iran, *T. indotineae* appears to be endemic. Over the past few years, a shift has occurred in skin infections from *T. interdigitale/mentagrophytes* toward *T. indotineae* in this country [23]. This species has also been linked with sexually transmitted dermatophytoses in recent reports from France and the USA [24,25,26]. The number of countries that have reported cases of *T. indotineae* infections is steadily increasing. Although the presence of *T. indotineae* is not comparable to the epidemic levels observed in India and only sporadic cases without large clusters have been reported so far, the potential for this epidemic to spread to additional countries in the near future should be taken into account. In Europe, cases have been reported from France [27,28], Switzerland [18,29,30], Greece [31], Denmark [32], Italy [33], United Kingdom [34], and Germany [35,36]. Outside Europe, *T. indotineae* has also been isolated in the USA [37,38], Brazil [39], China [40], Vietnam [41], Canada [42] among other regions. The majority of the *T. indotineae* strains described exhibited elevated terbinafine MICs and were associated with mutations in the squalene epoxidase (*SQLE*) gene. These cases were currently imported from India and neighboring countries (Pakistan, Bangladesh, Nepal, etc.); however, local transmission cannot be excluded, as autochthonous cases have been reported in several countries outside India [25,42,43,44]. A recent review of the literature on the emergence of terbinafine-resistant *T. indotineae* in Europe confirmed the evidence of human-to-human transmission [45]. In this context, the Belgian National Reference Center for Mycoses (BNRCM) initiated a nationwide study to assess the epidemiology of terbinafine resistance in Belgium. Strains presumed to be *T. indotineae*, *T. interdigitale*, and *T. mentagrophytes* isolated from skin samples and collected from multiple laboratories across Belgium (North and South regions), were thoroughly characterized for terbinafine resistance using both phenotypic and genotypic methods, including whole-genome sequencing.

## 2. Materials and Methods

### 2.1. Samples

All Belgian laboratories were invited to participate to the present study. Positive cultures from skin samples presumed to be *T. interdigitale*, *T. mentagrophytes*, or *T. indotineae* could be sent to the National Reference Center for Mycoses at the CHU of Liège. Comprehensive information about the study and its inclusion criteria was communicated to all Belgian laboratories through Sciensano, the National Institute of Public Health. Inclusion criteria were defined as followed: all Belgian laboratories could send every positive culture they isolated in their routine work from skin samples that was identified as *T. interdigitale/mentagrophytes/indotineae*. All positive cultures received at the NRC, were then sub-cultured on Sabouraud medium + cycloheximide (Biomérieux, Craponne, France) and incubated at 30 °C until growth.

### 2.2. Screening Method “Derma-Check”

Terbinafine resistance was initially screened using a custom agar-based dilution method that we called Derma-Check. Laboratory-grade terbinafine powder (Merck, Darmstadt, Germany) was dissolved in sterile dimethyl sulfoxide, and stock solutions at a concentration of 3.6 mg/mL were prepared and stored in aliquots at −80 °C. Dilutions were performed to achieve final terbinafine concentrations 0.05 µg/mL, 0.1 µg/mL, and 0.2 µg/mL in autoclaved Sabouraud dextrose agar medium (Merck, Germany). A growth positive control well without terbinafine was also included on the plate. The prepared medium was poured into four-well plates and allowed to cool. A dermatophyte suspension was prepared at a concentration of 0.5 McFarland units using a spectrophotometer (BioMérieux, France). A volume of 25 µL of the dermatophyte suspension was added to each well. The plate was then incubated at 30 °C for 98 h or until visible growth occurred on the positive control well. Resistance was suspected when a growth was observed on wells with 0.1 and/or 0.2 µg/mL of terbinafine. Strain IHEM 28378 (R to terbinafine) and IHEM28388 (S to terbinafine) have been used as quality controls.

### 2.3. EUCAST E.Def 11.0 Microdilution Plates

MICs were determined in parallel using the EUCAST E.Def 11.0 microdilution method, following established guidelines [46]. Briefly, inoculum suspensions were filtered through a sterile 11 µm pore diameter filter (Merck, Germany) and diluted 1:10 in sterile distilled water to achieve a final concentration of 2–5 × 10^5^ conidia/mL. The suspension was supplemented with chloramphenicol solution at a final concentration of 2 µL/mL from a 50 mg/mL stock solution (dissolved in ethanol) (Merck, Germany) and cycloheximide at 6 µg/mL from a 100 mg/mL ready-made stock solution (Merck, Germany). The final terbinafine concentration range after inoculation was 0.008–8 µg/mL. MICs were read at a 50% inhibition endpoint by spectrophotometry (490 nm) per EUCAST E.Def 11.0; MIC50 were then computed across isolates No EUCAST breakpoints are available for dermatophytes. Interpretation for all three species *T. interdigitale/mentagrophytes/indotineae* was based on the EUCAST tentative ECOFF value for *T. indotineae* (v3.0). For *T. interdigitale/mentagrophytes*, interpretation will consider WT and non-WT population rather than resistant or susceptible status for better accuracy. The strain SSI-9363 was used as quality control for every batch of handmade plates.

### 2.4. Whole Genome Sequencing

DNA extraction was conducted using the DNeasy UltraClean Microbial Extraction Kit (Qiagen, Hilden, Germany). Fungal material was collected and pretreated through bead beating, heat shock, and both chemical and enzymatic lysis. Specifically, fungal cultures grown in liquid Sabouraud medium were transferred into a PowerBead Tube (Qiagen, Germany) containing PowerBead solution and SL buffer supplied in the DNeasy UltraClean Microbial Extraction Kit. Heat shock was applied by placing the tube in liquid nitrogen for 1 min, followed by immersion in a 56 °C water bath for 1 min. Bead beating was performed for 10 min using a vortex mixer (VWR, Radnor, PA, USA). This cycle of heat shock and bead beating was repeated three times. DNA extraction was then completed following the manufacturer’s protocol using the DNeasy UltraClean Microbial Extraction Kit. The DNA yield was quantified using a NanoDrop 1000 spectrophotometer (ThermoFisher Scientific, Waltham, MA, USA). The extracted DNA was sequenced using the whole genome shotgun (WGS) method with Illumina sequencing (Illumina Inc., San Diego, CA, USA). Paired-end libraries were prepared from genomic DNA using the DNA PCR-Free Library Prep kit (Illumina Inc., USA) in accordance with the manufacturer’s instructions, requiring a minimum of 2 μg of genomic DNA per sample for library preparation.

The paired-end Illumina libraries were sequenced on the Illumina NovaSeq 6000 platform (Illumina Inc., USA) generating paired-end 2 × 150 bp reads. Raw read quality was assessed with FastQC V0.12.1 [47] and summarized with MultiQC V1.28 [48]. Reads were then filtered with fastp V0.20.1 [49] using default parameters (Phred quality threshold—q 15; maximum unqualified base proportion—u 40) with default adapter detection/trimming enabled [47,48,49]. These reads were then assembled de novo using SPAdes V 3.15.4 with the “careful” option enabled, including read error correction via the built-in BayesHammer module [50,51]. Genomes characteristics as coverage N50, genome size and contig count histogram are represented in Figure S3. For phylogenomic analysis, the tool used to generate genome alignments against reference genomes is REALPHY(https://realphy.unibas.ch/realphy/, accessed on 12 January 2025) [52]. All genomes included in the analysis, as well as secondary references were fragmented into 100 bp segments (all possible subsequences) and aligned to each reference genome. An alignment was then created for each reference, containing only “complete” positions, meaning orthologous positions present across all genomes. The alignments are combined so that each position of the references appears only once in the merged alignment. A phylogenetic tree is subsequently constructed using RAxML, (on Geneious Prime version 2022.2) with phylogeny inferred by the maximum likelihood method under the GAMMA-GTR model of evolution and 100 bootstrap replicates, identical isolates were not collapsed and tree rooting was performed using *M. canis* CBS 113480 strain [53]. The tree was then visualized using iTOL V7.2.1. The reference strains, TIMM20115 (JAJVHI000000000.1), TIMM20116 (JAJVHK000000000.1), TIMM20117 (JAJVHH000000000.1), TIMM20118 (JAJVHJ000000000.1), TIMM 20119 (JAJVHG000000000.1), IHEM28388 (JBKYKT000000000.1), IHEM28378 (JBKYKX000000000.1), IHEM28382, IHEM28384 (JBKYKU000000000.1), IHEM28380 and IHEM 28379 (JBKYKW000000000.1) were used as reference for *T. indotineae* identification. The reference strains IHEM4203, D15P127 (QQSR00000000.1) were used as reference for *T. mentagrophytes*. The strain MR816 (AOKY01000019.1) was used as reference for *T. interdigitale*. For SQLE analysis, the sequence was extracted from the WGS data using tBLASTn https://blast.ncbi.nlm.nih.gov/Blast.cgi, accessed on 14 January 2025)with reference sequence BAL48859.1. The ITS sequence was extracted from WGS data using BLASTn https://blast.ncbi.nlm.nih.gov/Blast.cgi, accessed on 27 June 2025) with reference KT253558 to define *T. mentagrophytes* type VII strains as described by Taghipour et al. [54]. Alignment method used was MAFFT. A phylogenetic tree was then generated using RAxML V8 on Geneious version 2022.2. The resulting tree was then visualized with iTOL. For the analysis of the ITS region to define type VII *T. mentagrophytes* and other ITS genotypes, the phylogeny was inferred by the maximum likelihood method under the GAMMA-GTR model of evolution and 500 bootstrap replicates. Tree rooting was performed using *T. mentagrophytes* MK 312891.

### 2.5. Statistics

Statistical analyses were conducted using SAS software version 9.4. The Kruskal–Wallis test was used to evaluate MIC values in relation to the presence of substitutions in the *SQLE* gene, while the Chi-Square test assessed differences in resistance patterns and clinical presentations across species. Additionally, a Scheffé Post Hoc test was performed to compare patient age distributions among the different species.

## 3. Results

### 3.1. Species Identification and Phylogenetic Classification

174 strains were collected during this study. 37 cultures were rejected; 9 because of culture contamination,7 for inappropriate origin (nail), 13 for inappropriate species (not *T. interdigitale/mentagrophytes/indotineae*), 4 showed no growth and 4 were duplicates.

**Figure 1:**
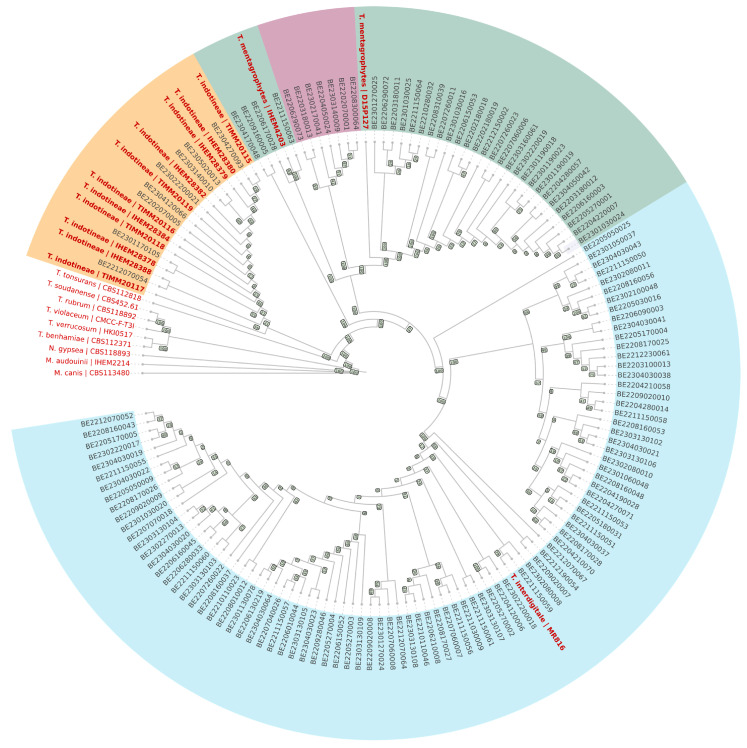
Species identification and phylogenomic classification of the 137 strains analyzed in this study based on whole genome sequencing data. Reference strains are indicated in red. *Trichophyton interdigitale* strains are shown in blue, *T. mentagrophytes* in green, with the type VII clade highlighted in purple. *T. indotineae* strains are depicted in orange. The dendrogram was constructed using RAxML and subsequently visualized with iTOL V7.2.1.

The identification of the 137 dermatophyte isolates was confirmed through whole-genome sequencing (WGS), followed by the construction of a maximum likelihood phylogenetic tree. This analysis revealed the presence of three well-defined clades. Specifically, eight isolates were assigned to *Trichophyton indotineae*, 91 to *Trichophyton interdigitale*, and 38 to *Trichophyton mentagrophytes*. The results are presented in Figure 1. Within the *T. mentagrophytes* clade, further analysis of the internal transcribed spacer (ITS) region enabled the identification of seven isolates as belonging to the *T. mentagrophytes* type VII subtype (highlighted in pink on the dendrogram, see also Figure S1). The remaining *T. mentagrophytes* isolates belonged to genotypes III* [24], and 5 strains were of undetermined genotype. Among *T. interdigitale*, 85 isolates corresponded to ITS genotype II, 4 to genotype I, one to genotype II* and 1 was of undetermined genotype. All *T. indotineae* isolates were identified as genotype VIII based on the ITS region (Figure S1).

### 3.2. Screening for Terbinafine Resistance Using the Agar Dilution Method

The screening for terbinafine resistance using the DermaCheck agar dilution method revealed positive growth in 7/137 strains, on Sabouraud dextrose medium wells containing 0.1 µg/mL or more of terbinafine. (See Supplemental Figure S2 for an example of plates reading).

### 3.3. Antifungal Susceptibility Testing by Microdilution (EUCAST E.Def 11.0)

All 137 strains included in the study were then assessed for antifungal susceptibility using the EUCAST E.Def 11.0 reference method. Minimum inhibitory concentrations (MICs) for terbinafine, itraconazole, voriconazole, and amorolfine were determined following this standardized protocol All results of the EUCAST E.Def.11.0 are illustrated in Table 1. All strains that grew on DermaCheck on wells with 0.2 µg/mL of terbinafine were confirmed to be with MICs ≥2 µg/mL for this drug by the EUCAST microdilution method. One strain showing the substitution L393S on SQLE showed a positive growth only in wells 0.05/0.1 µg/mL with a MIC of 2 µg/mL (see Table 2).

#### 3.3.1. Terbinafine Susceptibility Profile

Of the eight *Trichophyton indotineae* isolates tested, seven (87.5%) were classified as terbinafine-resistant based on EUCAST epidemiological cutoff values (ECOFFs). MICs ranged from 0.03 to 8 µg/mL, with a geometric mean of 4.41 µg/mL among resistant isolates. Among this group, 7/8 strains showed MIC ≥ 2 µg/mL. Within the *T. interdigitale* group, all strains were wild-type (WT), displaying MICs between 0.008 and 0.5 µg/mL and a corresponding geometric mean of 0.016 µg/mL. For *T. mentagrophytes*, six isolates showed MICs exceeding the ECOFF by 1–2 log_2_ dilutions. The MICs for this species spanned 0.008 to 0.5 µg/mL. Geometric means were 0.35 µg/mL for these putatively non-WT strains and 0.047 µg/mL for WT ones. All 6 non-WT strains were of genotype III*.

#### 3.3.2. Itraconazole Susceptibility Profile

Among the *T. indotineae* isolates, one strain (12.5%) was resistant to itraconazole, with a MIC of 4 µg/mL. This strain was also resistant to terbinafine (MIC = 4 µg/mL). The overall MIC range was 0.008–4 µg/mL, and the geometric mean for susceptible strains was 0.054 µg/mL. All *T. interdigitale* isolates showed a WT profile, with MICs ranging from 0.008 to 0.25 µg/mL and a geometric mean of 0.035 µg/mL. In the *T. mentagrophytes* group, two out of 38 isolates (5.2%) showed MICs higher than the ECOFF, MIC values ranged from 0.03 to 1 µg/mL. The geometric means were 0.116 µg/mL and 1 µg/mL for WT and potentially non-WT isolates, respectively.

#### 3.3.3. Voriconazole Susceptibility Profile

No resistance was observed among *T. indotineae* isolates, with MICs between 0.06 and 1 µg/mL and a geometric mean of 0.5 µg/mL. Similarly, all *T. interdigitale* strains were WT, with MICs in the 0.03–1 µg/mL range and a geometric mean of 0.179 µg/mL. In *T. mentagrophytes*, two isolates (5.2%) had MICs above the EUCAST ECOFF. MICs ranged from 0.125 to 2 µg/mL, with geometric means of 0.53 µg/mL for WT strains and 2 µg/mL for those exceeding the ECOFF.

#### 3.3.4. Amorolfine Susceptibility Profile

All *T. indotineae* strains appeared susceptible to amorolfine, with MICs ranging from 0.008 to 0.5 µg/mL and a geometric mean of 0.056 µg/mL. Among *T. interdigitale* isolates, 3 strains (3.3%) exhibited elevated MICs above the EUCAST-defined ECOFF. The geometric mean was 0.12 µg/mL for WT strains and 1 µg/mL for those potentially resistant. In *T. mentagrophytes*, 4 isolates (10.5%) also displayed MICs exceeding the ECOFF. The MIC range for this species was 0.125–1 µg/mL, with geometric means of 0.37 µg/mL for WT strains and 1 µg/mL for those classified as potentially non-WT.

### 3.4. Squalene Epoxidase Substitutions and Their Association with Terbinafine Resistance

Given the availability of whole genome sequencing data, particular attention was paid to the *squalene epoxidase* (SQLE) sequence. Several amino acid substitutions were identified in this region, some of which were associated with terbinafine resistance. Among the seven *Trichophyton indotineae* isolates classified as resistant to terbinafine, four harbored the F397L substitution in the SQLE. All four exhibited minimum inhibitory concentrations (MICs) ≥ 2 µg/mL for terbinafine. One strain harboring the F397L substitution was resistant to both terbinafine (MIC = 4 µg/mL) and itraconazole (MIC = 4 µg/mL). Two additional terbinafine resistant strains carried the L393F substitution and displayed MICs = 8 µg/mL, while one strain presented the L393S substitution with a MIC of 2 µg/mL. All these seven strains wearing substitutions on SQLE, grew on DermaCheck plates in well containing 0.2 µg/mL of terbinafine. A statistically significant difference in MICs values was observed between wild-type (considering all three dermatophytes species) and SQLE-mutated strains at positions F397L + L393F + L393S (*p* < 0.0001). Interestingly, one *T. indotineae* isolate susceptible to terbinafine showed the A448T substitution. Among the molecularly confirmed cases, the terbinafine resistance rate in our study was 5.12% (7 out of 137 strains). Within the *T. mentagrophytes* group, the K276N substitution on SQLE was identified in 29 out of 38 isolates (76%). Among these, six strains exhibited MICs for terbinafine that exceeded the EUCAST ECOFF (see Table 1). These findings are summarized in Table 2.

### 3.5. Impact of K276N on Antifungal Susceptibility

To further explore the potential role of the K276N substitution on SQLE, we compared antifungal susceptibility profiles between *T. mentagrophytes* strains harboring this substitution (n = 29) and wild-type (WT) strains lacking it (n = 9). Minimum inhibitory concentrations (MICs) for terbinafine, itraconazole, voriconazole, and amorolfine were assessed in both groups. A statistically significant difference in terbinafine MICs values was observed between the two groups: WT strains exhibited a geometric mean (GM) MIC of 0.026 µg/mL, whereas K276N strains showed a higher GM MIC of 0.085 µg/mL (*p* = 0.002). A similar trend was found for itraconazole, with GM MICs of 0.076 µg/mL for WT and 0.15 µg/mL for K276N strains (*p* = 0.03). For voriconazole, WT strains had a GM MIC of 0.29 µg/mL compared to 0.65 µg/mL for K276N strains (*p* = 0.003). Finally, in the case of amorolfine, the GM MIC was 0.25 µg/mL for WT strains and 0.43 µg/mL for K276N strains, also representing a statistically significant difference (*p* = 0.017). These observations are illustrated in Table 3.

### 3.6. Epidemiological Characteristics of Collected Dermatophyte Strains

**Figure 2:**
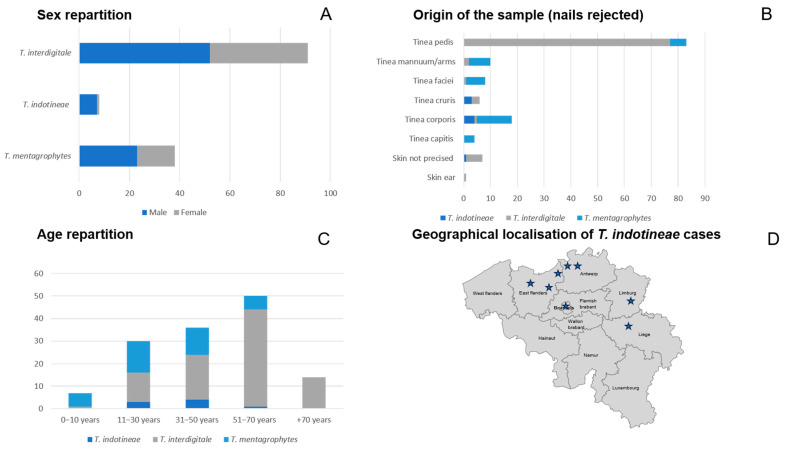
Epidemiological Characteristics of Collected Dermatophyte Strains. (**A**) Distribution of patient sex among the different species, (**B**) Clinical manifestations associated with each species, (**C**) Age distribution of patients by species, (**D**) Geographical distribution of *T. indotineae* cases in Belgium. Stars represent the localization of the case in Belgium. (Map adapted from https://fr.m.wikipedia.org/wiki/Fichier:Belgique_vierge.svg, accessed on 12 January 2025).

We next investigated the epidemiological profiles of the collected *Trichophyton* isolates. It should be acknowledged that this dataset presents an unequal distribution of lesion sampling, with tinea pedis cases being predominant. We could observe that *T. indotineae* infections predominantly affected young adults, with patient ages ranging from 18 to 56 years (mean + SD = 35.25 years ± 12.1). *T. mentagrophytes* was found across a broader age spectrum, including young children, with the 11–30-year age group most commonly affected (mean + SD = 31.3 years ± 17.5). In contrast, *T. interdigitale* was more frequently isolated from older individuals (mean + SD = 53 years ±18.6) compared to *T. indotineae* and *T. mentagrophytes*, particularly those aged 51–70 years (*p* < 0.0001), although cases were observed across all age groups. Regarding sex distribution, *T. indotineae* infections were strongly associated with male patients, accounting for 7 out of 8 cases. The gender distribution was more balanced for *T. interdigitale* (52 males vs. 39 females) and *T. mentagrophytes* (23 males vs. 15 females). Analysis of the anatomical origin of the isolates revealed species-specific patterns. *T. interdigitale* was primarily associated with tinea pedis (*p* < 0.0001). *T. mentagrophytes* was isolated from a variety of body sites including the face, arms, hands, inguinal region, trunk, and scalp. In contrast, *T. indotineae* was most commonly linked to tinea corporis and tinea cruris (*p* < 0.0001). All strains that showed MICs > 0.125 µg/mL for terbinafine were associated with tinea corporis/cruris. Geographically, *T. indotineae* cases were distributed across Belgium, with six cases reported in Flanders, one in Brussels, and one in Wallonia. Among these, six infections were identified as imported: two each from India, Nepal, and Bangladesh. The travel history of the remaining two cases was unknown. These results are shown in Figure 2.

### 3.7. Type VII T. mentagrophytes: Epidemiological and Resistance Profile

Phylogenomic/phylogenetic analysis identified seven isolates within the *T. mentagrophytes* clade as belonging to genotype VII. The majority of these cases occurred in male patients (6/7), with an age range of 28 to 59 years (mean = 36.1 years). Six of the seven isolates carried the K276N substitution on SQLE. All type VII isolates were phenotypically susceptible to terbinafine, although several displayed MICs values at the epidemiological cutoff value (ECOFF) established by EUCAST for *T. indotineae* (=0.125 µg/mL). Notably, one isolate exhibited an elevated MIC for itraconazole, exceeding the ECOFF, and another showed a MIC of 2 µg/mL for voriconazole also above the established ECOFF. This subtype was isolated from lesions located on the face (*tinea faciei*), body (*tinea corporis*), scalp (*tinea capitis*), thighs, and buttocks. Two cases involved multiple sites, including combinations of tinea corporis with arm involvement and tinea capitis with arm involvement. These results are illustrated in Table 4.

## 4. Discussion

This study represents the first national investigation in Belgium on dermatophytosis caused by members of the *Trichophyton mentagrophytes* series, with a specific focus on terbinafine resistance. Our whole-genome sequencing (WGS) analysis confirmed that *T. indotineae* is a distinct species, genetically differentiated from *T. mentagrophytes* and *T. interdigitale*. This finding aligns with previous studies, including that of Taghipour et al. [54], which demonstrated that *T. indotineae* (formerly referred as *T. mentagrophytes* type VIII) forms a separate clade based on analysis of the ITS region. In their 2020 publication, Kano et al. [7]. Formally described *T. indotineae* as a new species (*sp. nov.*) based on phylogenetic analysis and biochemical tests, which demonstrated that these strains clustered distinctly with Indian isolates and were clearly separated from the *T. interdigitale* clade [7].

All 137 strains included in our study were tested for antifungal susceptibility to terbinafine, voriconazole, itraconazole, and amorolfine. As expected, our findings confirmed that in general, *T. indotineae* strains exhibit high minimum inhibitory concentrations (MICs) for terbinafine. Based on the EUCAST-defined epidemiological cut-off values (ECOFFs), 7 out of 8 *T. indotineae* strains were classified as resistant to terbinafine, with MICs equals or exceeding 2 µg/mL.

In every case, elevated MICs were associated with substitutions on the squalene epoxidase (SQLE), in line with previous studies. Specifically, the F397L substitution was identified in four strains, while L393F and L393S were detected in two and one strain(s), respectively. These substitutions have been previously linked to disruption of terbinafine binding to its target, due to alterations in the size and shape of the drug’s hydrophobic binding pocket [5,15,16,17,18,20,21].

The only *T. indotineae* strain that remained susceptible to terbinafine harbored the A448T substitution. This particular substitution has been documented in the literature and is commonly associated with susceptibility to terbinafine, as it is located outside the terbinafine binding site and does not interfere with the drug’s inhibitory action on its target enzyme [27,55]. De Paepe et al. [56] reported that among twenty *T. indotineae* strains isolated from German patients between 2016 and 2019, nine carried the A448T substitution, and all of these were susceptible to both terbinafine and azoles [56]. However, some studies have linked this substitution to itraconazole resistance, and when it occurs in combination with F397L or L393F, it is often associated with resistance to both terbinafine and itraconazole [9,19,57,58]. In our study, the strain carrying this substitution did not exhibit any resistance to itraconazole.

All *T. interdigitale* strains were classified into WT population for terbinafine. Some *T. mentagrophytes* strains exhibited MICs values 1 to 2 log_2_ units above the ECOFF for terbinafine, despite the absence of detectable known substitutions on positions 393/397 of the SQLE. However, these strains harbored the K276N substitution. In the current literature, reports of terbinafine-resistant *T. mentagrophytes* var. *mentagrophytes* are rare, with only sporadic cases documented [31,37,59]. Considering all the *T. mentagrophytes* strains of our study, 29 out of 38 harbored the K276N substitution. Statistically significant differences were observed between wild-type and K276N strains, with the latter showing higher geometric mean (GM) MIC values for terbinafine, voriconazole, itraconazole, and amorolfine. Although this substitution has been previously reported in *T. mentagrophytes* isolates from Germany and China, it has not been associated with elevated MICs in those studies [20,43]. In a French study, this substitution was identified in *T. mentagrophytes* strains belonging to genotypes I/II, with MIC values of 0.25 µg/mL for both itraconazole and voriconazole. The authors did not report any significant increase in MIC values for terbinafine [28]. These findings suggest that while the K276N substitution has not been previously linked to elevated antifungal MICs, its association with significantly higher MIC values across multiple antifungal agents in our *T. mentagrophytes* strains warrants further investigation. This raises the possibility that K276N may contribute to subtle resistance mechanisms or serve as a marker of emerging phenotypic shifts within this species.

Based on molecularly confirmed resistant strains—identified through known resistance-associated substitutions on SQLE—our study found a terbinafine resistance rate of 5.12%, which is comparable to rates reported in other European countries. For instance, in France, Moreno Sabater et al. [26] documented a terbinafine resistance rate of 4.8% in a multicenter prospective study. In a Greek study focusing on 112 *Trichophyton* strains, the resistance rate to terbinafine was reported to be 8% [31]. A recent review of the literature performed by Ferreira et al. [45] in 2025 among a total of 378 publications, showed that 63 *T. indotineae* resistant to terbinafine have been reported from 8 different European countries [45]. These findings highlight that terbinafine resistance, while still relatively uncommon in Europe, is consistently present across various European countries, including Belgium. This underlines the importance of continued surveillance and molecular testing to promptly detect resistant strains and guide appropriate antifungal therapy.

With regard to azoles susceptibility, only one strain in our study showed resistance to both terbinafine and itraconazole, harboring the F397L substitution. These multidrug resistant *T. indotineae* have been already described in the literature [6,40]. Additionally, some *T. mentagrophytes* strains displayed MIC values 1 log_2_ unit above the ECOFF for azoles. Aside from *SQLE* gene sequencing, no further molecular analyses were conducted in our study to determine whether these strains possessed genetic markers associated with azole resistance, as previously reported in the literature [60,61,62,63]. These findings highlight the need for deeper molecular investigation beyond *SQLE* analysis to better understand the potential mechanisms underlying reduced azole susceptibility.

With regard to the epidemiological characteristics of our isolates, *T. indotineae* strains were predominantly identified in young male patients, with tinea corporis and tinea cruris being the most frequently associated clinical manifestations. Although our sample size was too limited to draw definitive conclusions, a review of the literature indicates that this species primarily affects young individuals. In a French study, seven reported cases involved four females and three males aged between 20 and 57 years [9]. Similarly, a prospective multicenter study conducted in the Czech Republic identified seven *T. indotineae* cases between 2018 and 2021, affecting four males and three females aged 23 to 44 years. Interestingly, three of these Czech cases presented as tinea pedis—a clinical form that is rarely reported in association with this species [59].

Genomic analysis enabled the identification, for the first time in Belgium, of seven *T. mentagrophytes* strains classified as type VII subtypes showing that this subtype also circulates in Belgium. These infections primarily affected young male patients and presented with diverse clinical manifestations, sometimes involving the thighs and buttocks. This subtype was initially described in association with sexually transmitted dermatophytosis in travelers returning from Southeast Asia, including Thailand, often following intensive sexual activity [64,65,66]. Transmission of this dermatophyte during sexual intercourse has been frequently reported also in Europe. Recent studies have emphasized that this subtype predominantly affects specific populations, particularly men who have sex with men [67,68,69]. In the current study, no information about the sexual orientation of the patients was available. Although resistance patterns in this species have been rarely reported, our study highlights the importance of monitoring susceptibility to terbinafine and azoles in this subspecies in order to prevent potential treatment failures. The identification of *T. mentagrophytes* type VII strains in Belgium marks a notable emergence of this genotype in Europe. Although the sexual orientation of patients in our study was not documented, the demographic profile and clinical presentations align with patterns described in the literature, particularly among young men with pubogenital involvement. These findings call for heightened clinical vigilance and the inclusion of *T. mentagrophytes* type VII in the differential diagnosis of sexually transmitted dermatophytoses, especially in at-risk population.

## 5. Conclusions

This study represents the first in-depth genomic and antifungal susceptibility analysis of isolates of the *Trichophyton mentagrophytes* series in Belgium, offering valuable insights into species distribution, resistance patterns, and emerging epidemiological trends. *T. indotineae* was clearly identified as a distinct species exhibiting terbinafine resistance, consistently associated with known SQLE substitutions. The identification of *T. mentagrophytes* type VII, predominantly in young male patients with sometimes pubogenital involvement, suggests a potentially expanding route of transmission in Europe of this sexually transmitted subspecies. Although information on patients’ sexual orientation was not available, the clinical presentations observed align with patterns reported in the literature, underscoring the need for increased clinical awareness among at-risk populations. Overall, our findings emphasize the importance of routine antifungal susceptibility testing, molecular surveillance of resistance mechanisms, and heightened diagnostic vigilance to ensure effective management of dermatophytoses in the context of evolving resistance and transmission dynamics.

## Figures and Tables

**Table 1:** MICs, resistance rates and geometric means for four antifungals (terbinafine, itraconazole, voriconazole and amorolfine) for the 137 *Trichophyton* species using the EUCAST E.Def.11.0 microdilution method.

**Terbinafine (Ecoff: 0.125 µg/mL)**				
Species	Range Terbinafine MIC (µg/mL)	Resistance Rate	Geometric Mean S/WT Strains (µg/mL)	Geometric Mean R/Non-WT Strains (µg/mL)
*T. indotineae*	0.03–8	7/8 (87.7%)	0.03	4.41
*T. interdigitale*	0.008–0.125	0/91 (0%)	0.016	NA
*T. mentagrophytes*	0.008–0.5	6/38 (15.8%)	0.047	0.35
**Itraconazole (Ecoff: 0.25 µg/mL)**				
Species	Range itraconazole MIC (µg/mL)	Resistance rate	Geometric mean S/WT strains (µg/mL)	Geometric mean R/non-WT strains (µg/mL)
*T. indotineae*	0.008–4	1/8 (12.5%)	0.054	4
*T. interdigitale*	0.008–0.25	0/91 (0%)	0.035	NA
*T. mentagrophytes*	0.03–1	2/38 (5.2%)	0.116	1
**Voriconazole (Ecoff: 1 µg/mL)**				
Species	Range voriconazole MIC (µg/mL)	Resistance rate	Geometric mean S/WT strains (µg/mL)	Geometric mean R/non-WT strains (µg/mL)
*T. indotineae*	0.06–1	0/8 (0%)	0.5	NA
*T. interdigitale*	0.03–1	0/91 (0%)	0.179	NA
*T. mentagrophytes*	0.125–2	2/38 (5.2%)	0.53	2
**Amorolfine (Ecoff: 0.5 µg/mL)**				
Species	Range amorolfine MIC (µg/mL)	Resistance rate	Geometric mean S/WT strains (µg/mL)	Geometric mean R/non-WT strains (µg/mL)
*T. indotineae*	0.008–0.5	0/8 (0%)	0.056	NA
*T. interdigitale*	0.016–1	3/91 (3.3%)	0.12	1
*T. mentagrophytes*	0.125–1	4/38 (10.5%)	0.37	1

MIC = Minimum Inhibitory Concentration; S = Susceptible; R = Resistant; NA = Not Applicable; WT = Wild-Type; non-WT = Non-Wild-Type.

**Table 2:** Squalene epoxidase (SQLE) substitutions and their association with terbinafine resistance.

DermaCheck Status 0.05/0.1/0.2 µg/mL TERB	SQLE Substitution	Number of Isolates (Species)	Susceptibility Profile	MIC TERB (µg/mL)
Growth (0.05/0.1/0.2 µg/mL) [4]	F397L	4 (*T. indotineae*)	R	2–8
Growth (0.05/0.1/0.2 µg/mL) [2]	L393F	2 (*T. indotineae*)	R	8
Growth (0.05/0.1 µg/mL)	L393S	1 (*T. indotineae*)	R	2
No growth [1]	A448T	1 (*T. indotineae*)	S	0.03
No growth [27]	K276N	29 (*T. mentagrophytes*)	WT, (*n* = 14) ≥ECOFF (*n* = 15)	<0.125 (*n* = 14) 0.125–0.5 (*n* = 15)

Abbreviations: R = Resistant; S = Susceptible; MIC = Minimum Inhibitory Concentration; TERB = Terbinafine; ECOFF = Epidemiological cut-off value; WT = wild-type.

**Table 3:** Impact of K276N on MICs values of different antifungals.

SQLE Substitution	TERB MIC Range (µg/mL)	TERB GM (µg/mL)	*p* Value	ITRA MIC Range (µg/mL)	ITRA GM (µg/mL)	*p* Value	VOR MIC Range (µg/mL)	VOR GM (µg/mL)	*p* Value	AMOR MIC Range µg/mL	AMOR GM µg/mL	*p* Value
K276N [27]	0.008–0.5	0.085	0.002	0.008–0.5	0.15	0.03	0.125–2	0.65	0.003	0/125–1	0.43	0.017
WT [9]	0.008–0.03	0.026		0.03–0.25	0.076		0.125–0.5	0.29		0.125–0.5	0.25	

GM = Geometric Mean; MIC = Minimum Inhibitory Concentration; TERB = Terbinafine; ITRA = Itraconazole; VOR = Voriconazole; AMOR = Amorolfine.

**Table 4:** Type VII *T. mentagrophytes*: Epidemiological and Resistance Profile.

Localization of the Lesion	SEX	Age	MIC TERB (µg/mL)	MIC ITRA (µg/mL)	MIC VOR (µg/mL)	MIC AMOR (µg/mL)	SQLE Substitution
Face	M	34	0.03	0.06	0.25	0.125	WT
Scalp and arm	M	28	0.03	1	1	0.5	K276N
Nose	M	33	0.06	0.125	1	0.5	K276N
Buttocks	M	30	0.125	0.25	1	0.5	K276N
Trunck + arm	M	40	0.125	0.125	2	1	K276N
Thigh	F	59	0.125	0.125	1	0.5	K276N
Trunck	M	29	0.125	0.125	1	0.25	K276N

MIC = Minimum Inhibitory Concentration; TERB = Terbinafine; ITRA = Itraconazole; VOR = Voriconazole; AMOR = Amorolfine; M = Male, F = Female; SQLE = Squalene epoxydase.

## Data Availability

Whole genomes of *T. indotineae* characterized in this study were deposited to Genbank under the bioproject ID: PRJNA1288206.

## Supplementary Materials

The following supporting information can be downloaded at: https://www.mdpi.com/article/10.3390/jof11090676/s1, Figure S1. Phylogenetic tree based on ITS regions generated using RAxML and visualized with iTOL V7.2.1. Type VII strains characterized in this study are highlighted in purple. Reference strains (in red) used for comparison were those described by Taghipour et al. [54]. Figure S2. Representative DermaCheck result illustrating suspected terbinafine resistance in strains from line 1 and 3; the strain in line 2 is susceptible. Row 1 is the growth control without terbinafine while row 2, 3, 4 contain respectively 0.05 µg/mL, 0.1 µg/mL and 0.2 µg/mL of terbinafine. Figure S3. Graphical representation of the coverage per isolate vs N50, the genome size distribution and contig counts for all the 137 genomes characterized by WGS during this study.
