# Supplementary material for: National Belgian Study on Terbinafine Resistance in Trichophyton interdigitale/mentagrophytes/indotineae (2022–2023): Epidemiology and Molecular Features
Source: J Fungi (Basel). 2025 Sep 13;11(9):676. doi: 10.3390/jof11090676 (PMC12470705; doi:10.3390/jof11090676)
Supplement: Supplementary file 1 [file jof-11-00676-s001.zip › jof-3837076-supplementary.pdf]

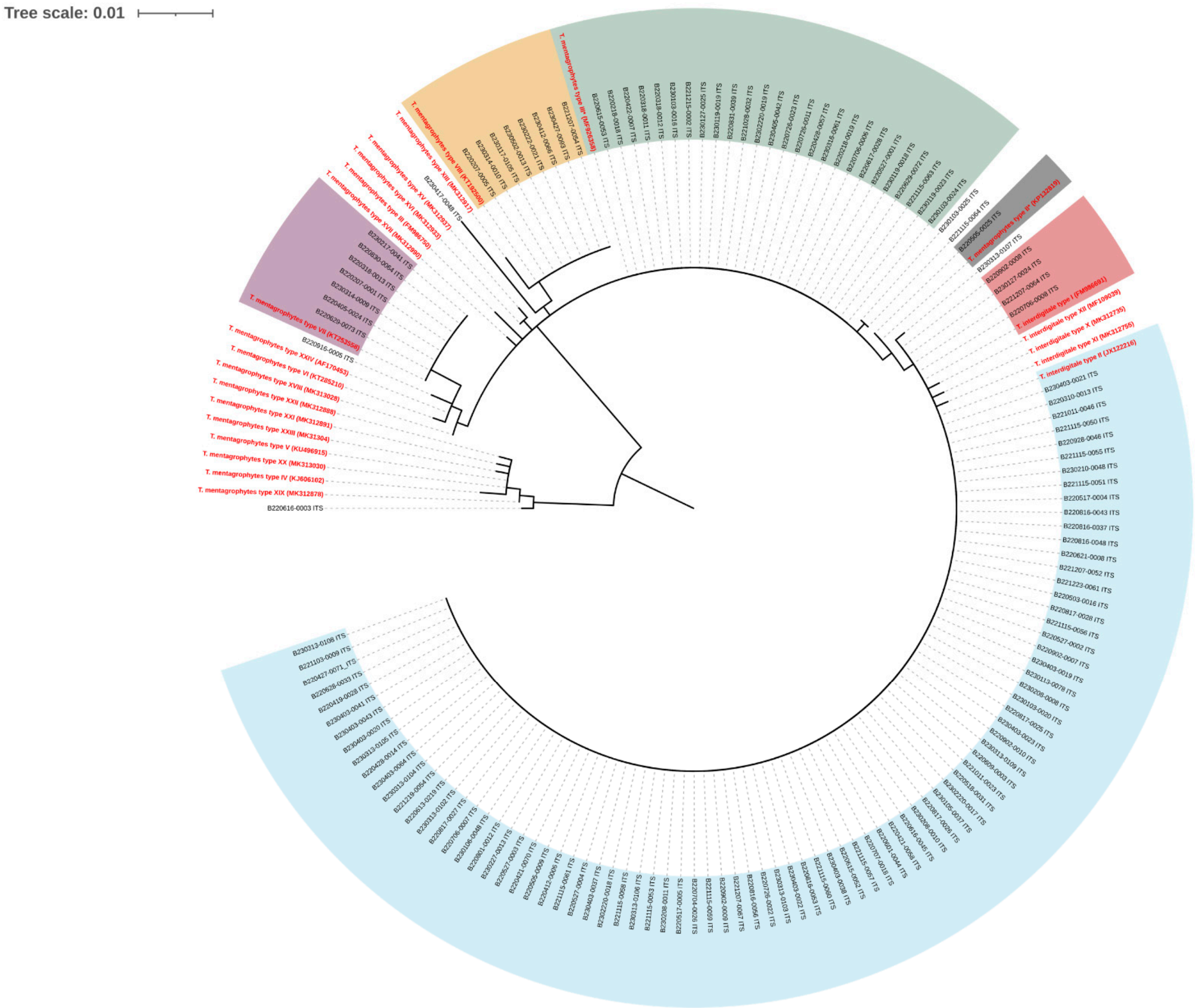

Supplemental Figure S1. Phylogenetic tree based on ITS regions generated using RAxML and visualized with iTOL V7.2.1. Type VII strains characterized in this study are highlighted in purple. Reference strains (in red) used for comparison were those described by Taghipour et al. [54].

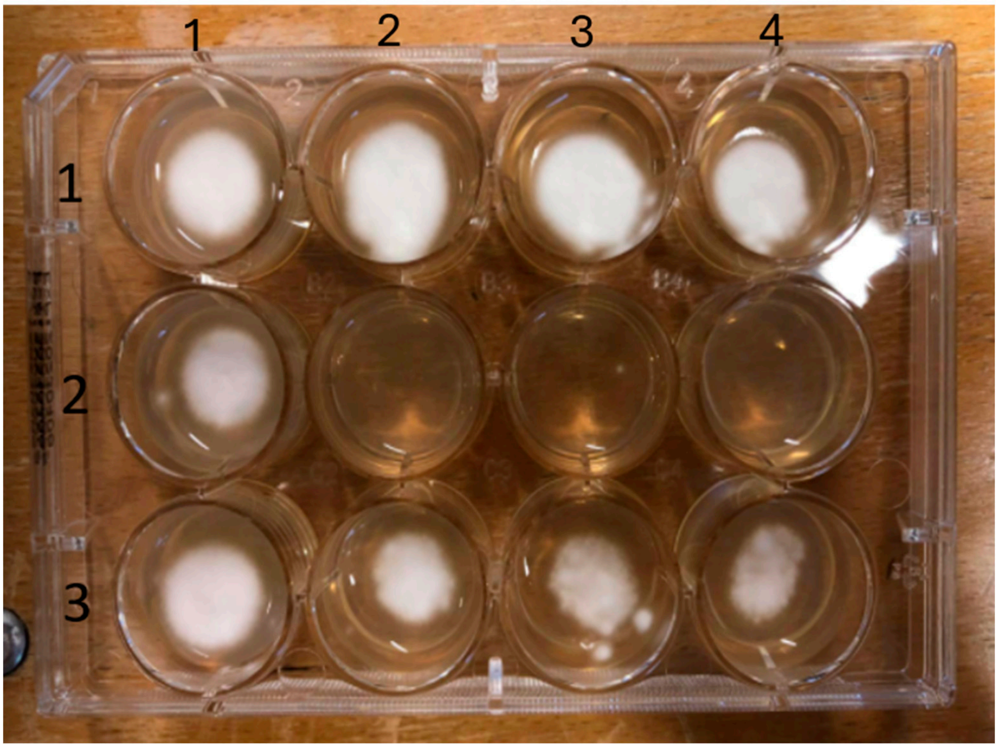

Supplemental Figure S2. Representative DermaCheck result illustrating suspected terbinafine resistance in strains from line 1 and 3; the strain in line 2 is susceptible. Row 1 is the growth control without terbinafine while row 2, 3, 4 contain respectively 0.05µg/mL, 0.1µg/mL and 0.2µg/mL of terbinafine.

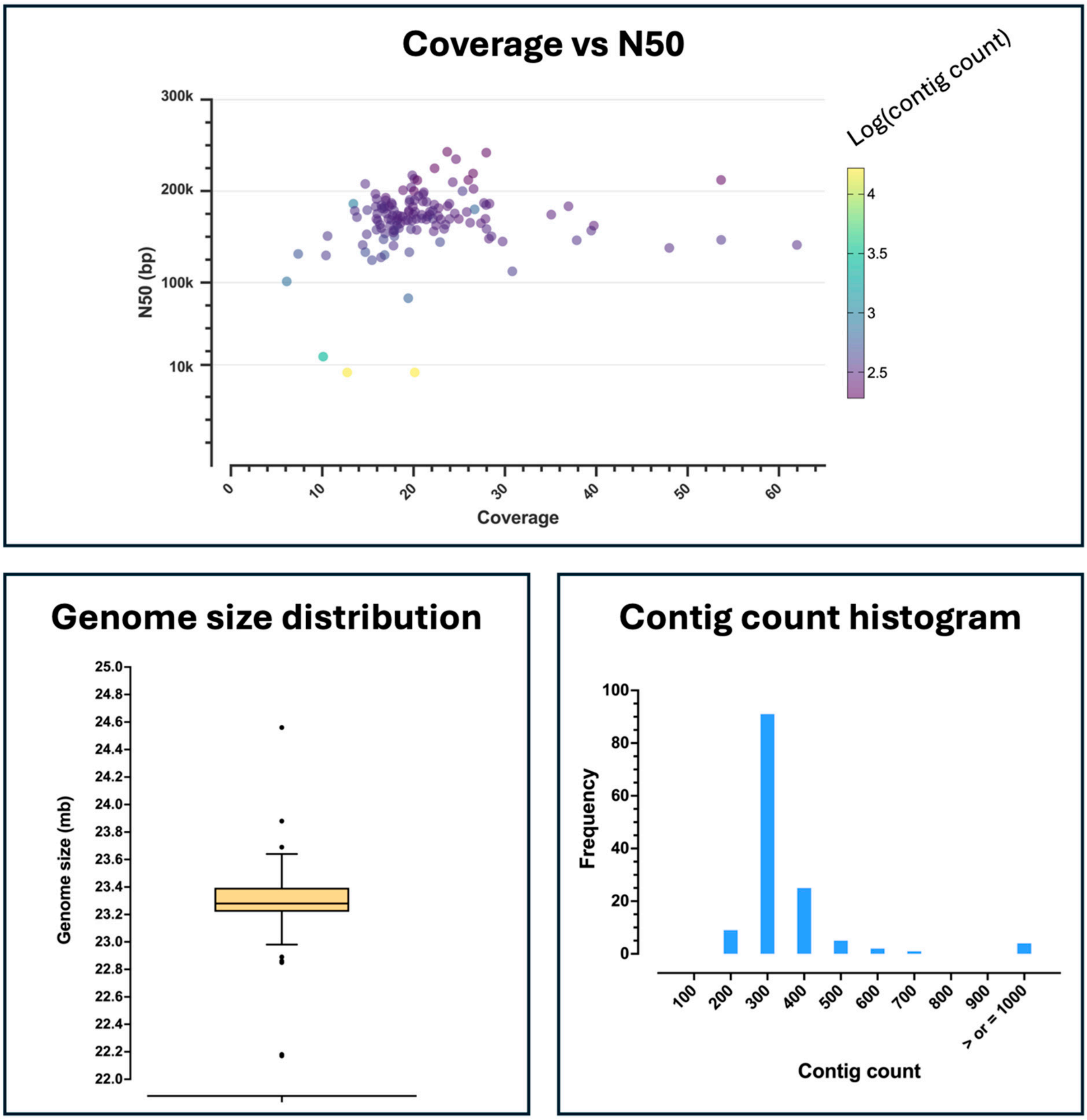

Supplemental Figure S3. Graphical representation of the coverage per isolate vs N50, the genome size distribution and contig counts for all the 137 genomes characterized by WGS during this study.
